# Radon Levels in Indoor Environments of the University Hospital in Bari-Apulia Region Southern Italy

**DOI:** 10.3390/ijerph15040694

**Published:** 2018-04-07

**Authors:** Luigi Vimercati, Fulvio Fucilli, Domenica Cavone, Luigi De Maria, Francesco Birtolo, Giovanni Maria Ferri, Leonardo Soleo, Piero Lovreglio

**Affiliations:** 1 Interdisciplinary Department of Medicine, Section of Occupational Medicine, University of Bari Aldo Moro Medical School, 70124 Bari, Italy; domenica.cavone@uniba.it (D.C.); luigi.demaria@uniba.it (L.D.M.); francesco.birtolo85@gmail.com (F.B.); giovannimaria.ferri@uniba.it (G.M.F.); leonardo.soleo@uniba.it (L.S.); piero.lovreglio@uniba.it (P.L.); 2 Regional University Hospital Policlinico—Prevention and Protection Service/Health Physics—A.O.U. Consortium Policlinico di Bari, 7014 Bari, Italy; fulvio.fucilli@policlinico.ba.it

**Keywords:** radon, university hospital, exposure, health care workers

## Abstract

Since 1988, the International Agency for Research on Cancer (IARC) has classified radon among the compounds for which there is scientific evidence of carcinogenicity for humans (group 1). The World Health Organization (WHO) recommends a reference radon level between 100 and 300 Bq/m^3^ for homes. The objective of this study is to measure the radon concentrations in 401 workplaces, different from the patient rooms, in 28 different buildings of the university hospital in Bari (Apulia region, Southern Italy) to evaluate the exposure of health care workers. Radon environmental sampling is performed over two consecutive six-month periods via the use of passive dosimeters of the CR-39 type. We find an average annual radon concentration expressed as median value of 48.0 Bq/m^3^ (range 6.5–388.0 Bq/m^3^) with a significant difference between the two six-month periods (median value: February/July 41.0 Bq/m^3^ vs. August/January 55.0 Bq/m^3^). An average concentration of radon lower than the WHO reference level (100 Bq/m^3^) is detected in 76.1% of monitored environments, while higher than 300 Bq/m^3^ only in the 0.9%. Most workplaces report radon concentrations within the WHO reference level, therefore, the risk to workers’ health deriving from occupational exposure to radon can be considered to be low. Nevertheless, the goal is to achieve near-zero exposures to protect workers’ health.

## 1. Introduction

Radon is a radioactive, colorless and odorless gas that is naturally generated by the decay of radium, produced by the transformation of uranium, which is in turn present in rocks, soil, water and building materials [1,2,3,4]. The most stable isotope is radon-222 which decays within a few days, emitting ionizing radiation of the alpha type and forming the so-called “decay products” that are themselves radioactive. Radon 222 is a product of the greatest importance in terms of the dose of natural radioactivity, because of its chemical characteristics that allow it to be spread in the atmosphere and be breathed by humans.

Indeed, radon-containing gas coming from the ground can penetrate buildings through the foundations, through cracks in the walls and through the hydraulic drainage systems according to a combination of the molecular diffusion principles described by Fick’s law and the gas diffusion described by Darcy’s law [5,6]. Other than emissions from the ground, important sources of radon are building materials, particularly clay and cement; moreover, the contributions of water for domestic use that comes from wells located in areas with high radioactivity and the combustion of gases for energy production in buildings might be other sources [2,3,4,5,6].

Therefore, radon tends to concentrate inside buildings in which the exchange of air is limited and in underground environments, such as basements and mines. Potentially high levels can even occur on the first floors of buildings, although in certain office-type buildings or buildings with elevators or installation shafts, radon can be similar in higher and lower floors probably due to the so-called “chimney effect” [7]. On the other hand, radon concentrations are low in outdoor environments due to its rapid dispersion [8]. This process is favored by the short half-life of radon, which is equal to 3.82 days.

The natural radioactivity produced by radon and its decay products represents an important source of exposure to ionizing radiation for humans. Among several international organizations, this exposure has stimulated a growing interest in the phenomenon of radioactivity in confined living and working environments in which the population spends most of its time, particularly in industrialized countries. The main effect of the inhalation of radon and its decay products on human health is lung cancer. Since 1988, the International Agency for Research on Cancer (IARC) has classified radon among the compounds for which there is certain scientific evidence of carcinogenicity to humans (group 1) [9]. Specifically, it has been estimated that radon is the main cause of the onset of pulmonary neoplasia after cigarette smoking [2]. The World Health Organization (WHO) notes that indoor radon exposure causes from 3% to 20% of lung cancer worldwide [2]. According to current estimates by the US Environment protection agency (EPA), each year approximately 21,000 lung cancer deaths in the United States are associated with radon exposure [4]. In 2012, 17% of lung cancer cases in Alberta were found to be attributable to residential radon exposure [10]. Occupational exposure to high concentrations of radon have also been demonstrated to increase the risk of lung cancer in nonsmokers [11].

The effects of radon on cancers other than lung cancer have also reported, [2,3,4,12,13]. For example, the incidence rates of chronic lymphocytic leukemia (CLL) among US states have been reported to be significantly correlated with the levels of residential radon (RR) [14]. Statistical associations of indoor radon levels with lung, stomach and brain cancers in women in Galicia have also been reported [15].

Following the classification of radon among carcinogens, many countries and international organizations have issued norms or recommendations for limiting exposure. The WHO recommends a reference level of 100 Bq/m^3^ for homes, and the International Commission for Radiological Protection has also recommended a level not exceeding 300 Bq/m^3^ [2,16,17].

In Italy, the regulation of radon exposure in the workplace was introduced in 2001 with Legislative Decree no. 241/00 [18] that implemented the Directive 29/96 Euratom, which modified and integrated Legislative Decree 230/95. With the aforementioned decrees, occupational exposures in underground workplaces, such as caves and tunnels, and in working areas that are highly likely to have high concentrations of radon (occasionally called radon-prone areas), became subject to the control of radon levels. The legislation states that if the average annual concentration of radon in the workplace exceeds the action level of 500 Bq/m^3^, the employer must implement remedial action to reduce the concentration. Afterwards, in line with Directive 2013/59/EURATOM, both the National Radon Plan (PNR) [19] and the Apulian Regional Law 30/16 (subsequently modified by the Regional law no. 36/2017) [20], has established that the reference limit level for the concentration of radon gas activity in closed environments of new buildings, in buildings intended for education and in buildings that are open to the public must not exceed 300 Bq/m^3^ in all premises of the building in question as measured as an annual average concentration.

The purpose of the study is to measure the radon concentrations in the working environments of the university hospital in Bari, considering the particular layout that involves the presence of many rooms belonging to different buildings but included in a restricted area, and compare the obtained results with the different reference limits.

## 2. Materials and Methods

**Figure 1:**
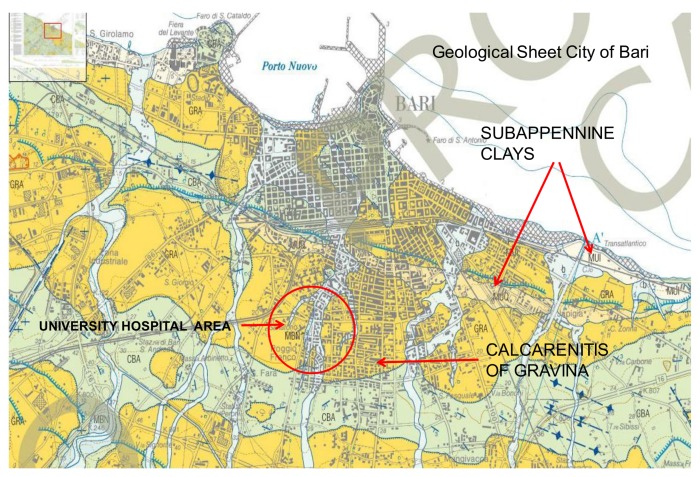
Geological sheet of the city of Bari.

The study covered the work environments of the consortium university hospital A.O.U.C. Policlinico di Bari, which is a hospital–university company located in the city of Bari (Apulia) that is entirely built on a base of calcarenite of gravina, which is clastic rock that is formed almost entirely of calcium carbonate (Figure 1). It has an extent of about 230 thousand square meters.

The monitoring was conducted during 2014–2015 and involved a total of 401 samples taken from the premises of the company, which are divided into approximately 28 buildings that contain 395 basements with floor levels placed approximately 3.5 m below road level and 6 upper basements with walking surfaces that are approximately 1 m from street level. Each studied environment is continuously occupied by workers (at least 6 h a day for 5 days a week). For each sample, a single card that identified the dosimeter code, the positioning date, the collection date and the identification of the local object of the measure was completed.

Environmental radon measures were collected for two consecutive six-month periods, i.e., the first period (February–July) and the second period (August–January) via the use of passive dosimeters of the CR-39 type detectors that respond to nuclear tracks. All dosimeters were placed at a height of approximately 2 m from the floor and at least 0.3 m away from external walls to exclude influence from Tn, heat sources, windows or other objects.

These dosimeters consist of a polyvinylchloride container that allows for the passage of radon into it until the level equilibrates in a short time with the concentration present outside. Inside, there is a plate of CR-39, which is a transparent allyl polymer that is sensitive to α radiation. Inside the dosimeter, the radon and its descendants emit α particles that ionize the detector’s solid matrix and break the atomic bonds along a perfectly straight path leaving tracks whose densities are proportional to the radon concentration. The density of nuclear tracks found per cm^2^ in each detector can be expressed in terms of radon concentration (measured in Bq/m^3^) with the aid of an appropriate calibration factor. The calibration constant that was established for the CR39 dosimeters was equal to one track per cm^2^ of exposure (0.41 Bq/m^3^), and the minimum detectable concentration was 10 Bq/m^3^ for a period of exposure of 3 months.

The calibration of the dosimeters was performed in the radon room in the National Agency for New Technologies, Energy and Sustainable Economic Development (ENEA) primary metrological institute. For each monitored environment, based on the effective monitoring duration for each period, the minimum detectable concentration was calculated with consideration of the minimum detectable exposure value indicated by the supplier of the dosimeters (CR-39) supplied by Tecnorad Verona Italy, which was provisionally set at a value of 25 KBq h/m^3^ divided by the actual duration of the measure. For the analysis of the dosimeters, the chemical development was performed with the POLITRACK automated optical measuring system, which was developed by the Mi.am Srl company in collaboration with the energy department of the Polytechnic University of Milan [21].

The results were interpreted according to the current national and regional regulations in compliance with the regional reference level (300 Bq/m^3^) [19,21] and according to the WHO criteria and the EPA reference value, dividing indoor radon concentrations into < 100 Bq/m^3^, 100–148 Bq/m^3^ and > 148 Bq/m^3^ [4].

Among the 401 examined environments, 42 premises, all of which were on basement floors, presented average annual concentrations of radon gas above 148 Bq/m^3^, the EPA reference value. These premises became the subject of a new inspection because, currently, some of them have been abandoned. The subjects of the new survey included 31 rooms, and, specifically, the cubage was determined, and information was acquired regarding the type and the cumulative wear of the flooring, the presence of air conditioners, radiators and windows and the degree of ventilation.

Statistical analyses were done using the SPSS program (version 14.0, Chicago, IL, USA). The variables were normalized with the log-transformation and analyzed using parametric tests, the level of significance was set at *p* < 0.01.

## 3. Results

We found an average annual radon concentration expressed as median value of 48.0 Bq/m^3^ with a minimum value of 6.5 Bq/m^3^ and a maximum value of 388 Bq/m^3^ (Table 1).

**Figure 2:**
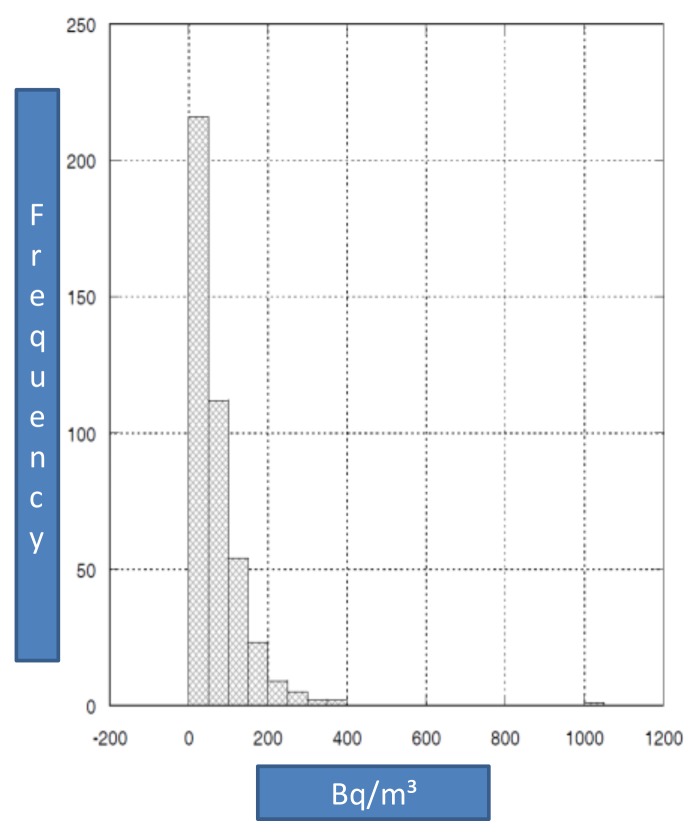
Distributions of the annual average radon concentrations expressed as Bq/m^3^.

Figure 2 presents the distribution of the average annual radon concentrations that were measured in the 401 monitored environments.

The concentration of radon gas was significantly higher in the first period (February–July) than in the second period (August–January) and a significant positive correlation was observed between the two periods (r = 0.788, *p* < 0.01). A median level of 35.8 Bq/m^3^ with a minimum value of 32 Bq/m^3^ and a maximum value of 147.5 Bq/m^3^ (Table 1) was found in the upper rooms, lower than that observed in the basement rooms.

All of the monitored environments exhibited annual average radon concentrations that were lower than the limit of 500 Bq/m^3^ that was established by Legislative Decree 241/00 [18], and only four environments (0.9%) exceeded the limit of 300 Bq/m^3^ set by the regional law (LR) no. 30/2016 (Table 2). Moreover, in 76.1% of the rooms, an average concentration of radon lower than the WHO reference level was detected (Table 2).

**Figure 3:**
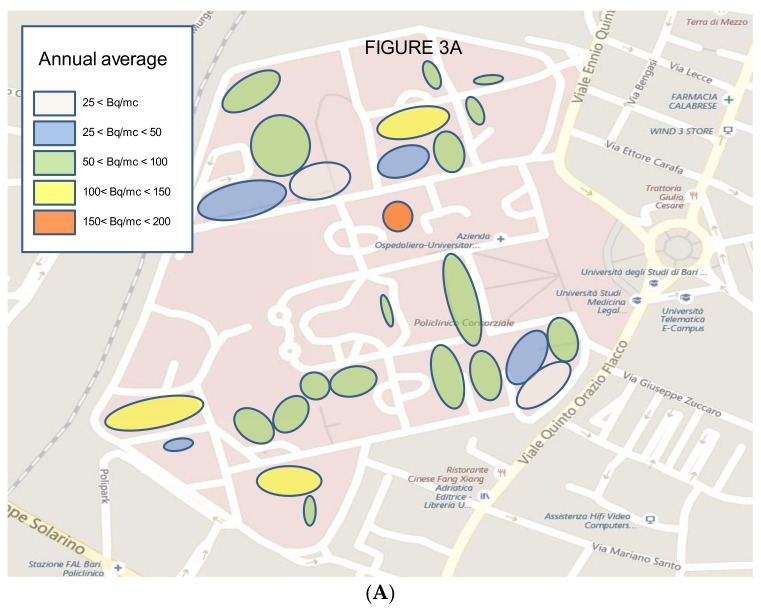

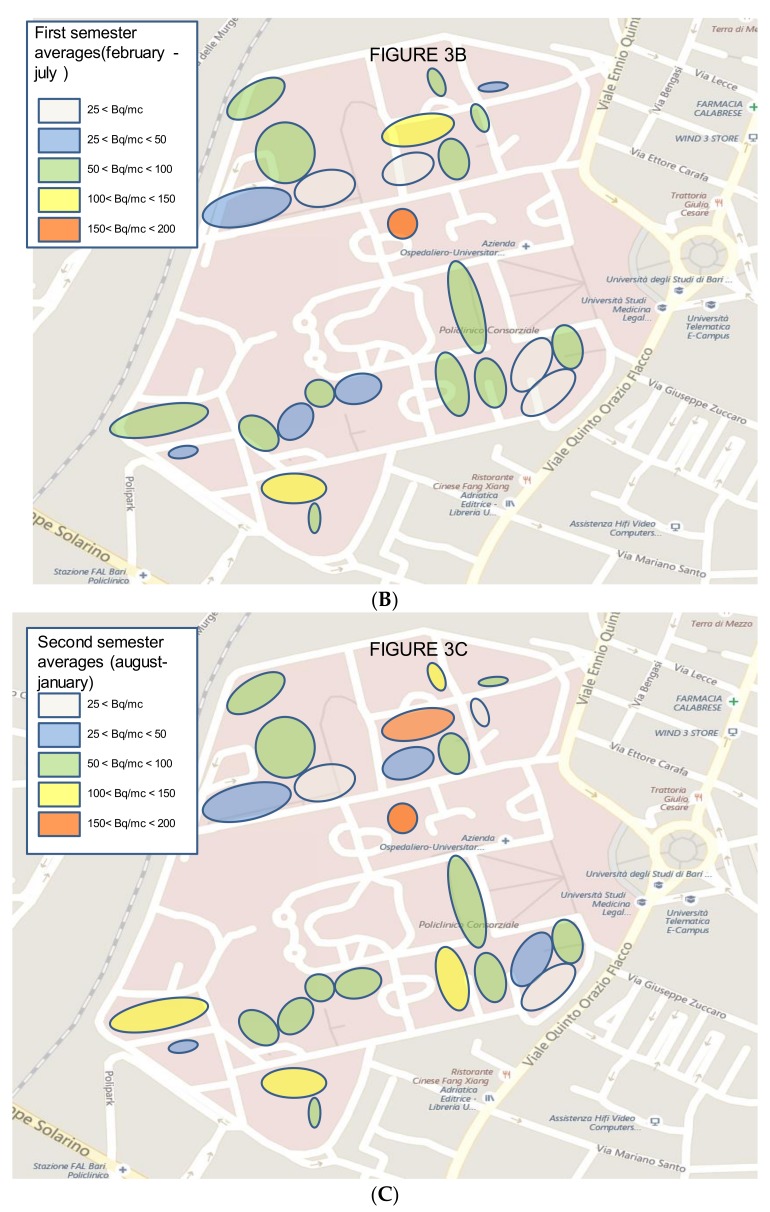
(**A**–**C**) Distributions of the radon concentrations in the different areas of the hospital as annual average and in the two periods, expressed as Bq/m^3^ (scale 1: 100).

Figure 3A presents the distribution of the average radon concentrations in the different areas of the hospital, while, in Figure 3B,C, the variations in the different buildings between the two periods are visible and highlighted by the different colors for the same site.

Among the 42 environments with annual average radon concentrations higher than 148 Bq/m^3^, all were basement floors, and 11 were renovated and completely modified after the measurements. A survey was performed in the remaining 31 environments, which were in 10 buildings (Pathological Anatomy, Gastroenterology, Neurology, Morgue, Dentistry, Otolaryngology, Infectious Diseases, Physical Medicine and Rehabilitation, Radiodiagnostics and Nursery). The examined environments had cubages between 23 and 239 m^3^; 23 of these (74.2%) had tile flooring that was in good condition in 10 rooms, with cracks or minimal fissures in 13 rooms. Six rooms had PVC tile floorings; in five of these rooms, cracks or minimal fissures were detected. One room had parquet flooring in good condition. Air conditioners were present in 26 rooms (83.9%), radiators in 16 rooms (51.6%) and windows in 21 rooms (67.7%). In these 31 rooms, the annual average radon concentrations was between 151 and 393 Bq/m^3^. No significant correlation was observed between annual average radon concentrations and the cubage of the 31 rooms. The highest concentration was measured in the only environment with parquet flooring in good condition, which had a cubage of 239 m^3^ and four windows that were opened for seven hours a day.

## 4. Discussion

All the monitored environments had radon concentration lower than the limit of 500 Bq/m^3^ that was established by Legislative Decree 241/00 [19], and only four environments (0.9%) exceeded the limit of 300 Bq/m^3^ set by LR no. 30/2016 [20]. Most of the rooms (76.1%) had radon concentrations that were within the reference value set by the WHO and 99.1% within the limit value set by the regional law. Therefore, the risk to the workers’ health deriving from occupational exposure to radon could be considered admissible according to the regional law, although, based on WHO standards, 23.9% of the workplaces exceeded standards and it should be considered that there is no threshold value below which radon does not have certain effects on human health [2].

The average concentrations of radon detected in our study are in line with the national figure of 75 Bq/m^3^, which was derived from a national survey of radon concentrations in homes performed between 1989 and 1994 by the Italian Higher Institute of Health (ISS) and the Environmental Protection Agency and Technical Services (APAT) [22].

The worldwide average radon concentration in indoor environments is slightly lower (approximately 39 Bq/m^3^) than the national average in Italian homes [2,22]. A recent study conducted in Apulia allowed for the estimation of a geometric mean radon concentration of 114 Bq/m^3^ across 311 homes. However, this value is significantly higher than the national average and appears to have been influenced by the large number of houses from the old construction period and structural deterioration of the houses that were included in the study [23].

Moreover, as expected, the average radon concentration found in the upper floors was lower than that found in the basement floors. This finding is in line with findings that have been reported in the literature [24,25] and is due to the manner in which radon penetrates into environments. Indeed, radon migrates from the ground into buildings by penetrating through cracks, wall–floor joints and passages of the thermal, electrical and hydraulic systems. Consequently, radon levels are generally higher in basement floors and are reduced in upper floors. Moreover, as expected, seasonal variations can significantly affect indoor radon concentration [26].

The figures underline how in a small area with an identical geological characterization, both in the same buildings and between different buildings at a limited distance from each other, in the order of some tens of meters, very different values were measured.

The later inspections that were performed in the 31 environments (all basements) that had critical radon values, revealed cubages between 23 and 239 m^3^, not correlated to radon concentrations. In 19 rooms (61%), the tile and PVC floors were worn out. In 10 rooms (32%), windows were absent, and only in five rooms (16%) there was no air conditioning system. In all of these rooms, the level of ventilation seemed to be insufficient. In the rooms in which values exceeding the limits were found, further investigations are necessary to identify the way in which radon is introduced (e.g., cracks in walls, floors, cavities, piping, cable routes for electrical systems, etc.), to analyze the impact of ventilation systems and air exchange rates and thermal retrofit works on the mechanisms governing indoor radon concentrations [27] and the most suitable procedures must be implemented to achieve compliance. At the end of the interventions, new measurements will be performed to verify the effectiveness of the interventions and to undertake remediation actions, first to improve building ventilation and if necessary radiation protection procedures initiated.

## 5. Conclusions

Most workplaces (76.1%) of the university hospital reported radon concentrations that are within both the WHO reference values and those set by the regional law. The risk to workers’ health deriving from occupational exposure to radon can be considered to be admissible according to the regional law. It should however be stressed that the regional law has limit values three times higher than WHO reference value.

Several studies have demonstrated a linear relationship, rather than a threshold relationship, between residential exposure to radon and lung cancer risk, which indicates that there is no safe radon level. The majority of radon-induced lung cancer deaths are caused by radon concentrations below the reference levels used here, and the general population is very frequently exposed to low levels of indoor radon. Therefore, the national and international exposure limit values may need to be revised. We will need to achieve near-zero exposures to protect public health.

## Figures and Tables

**Table 1:** Radon concentrations in the examined premises (Bq/m³).

	N.	Median	Range
Average annual levels	401	48.0	6.5–388.0
Semesters			
- February–July	401	41.0	5–538
- August–January	401	55.0	6–458
Workplace location			
- Basement premises	395	49.0	6.5–388.0
- Upper premises	6	35.8	32–147.5

**Table 2:** The levels of radon in relation to the World Health Organization WHO (100 Bq/m^3^), Environmental Protection Agency EPA (148 Bq/m^3^), regional law (LR) n.30/2016 (300 Bq/m^3^) reference values.

Radon Levels Concentration (Bq/m³)	Number of Rooms	%
< 100	305	76.1
100–148	54	13.5
149–300	38	9.5
> 300	4	0.9
